# The associations between serum trace elements and bone mineral density in children under 3 years of age

**DOI:** 10.1038/s41598-021-81501-3

**Published:** 2021-01-21

**Authors:** Ziyi Wu, Yuhao Yuan, Jian Tian, Feng Long, Wei Luo

**Affiliations:** Department of Orthopaedics, Xiangya Hospital, Central South University, Changsha, 410008 China

**Keywords:** Paediatric research, Metabolic bone disease, Malnutrition, Orthopaedics, Metals

## Abstract

We examined the associations of age and serum magnesium, iron, lead, copper, and zinc levels with bone mineral density (BMD) in 2412 children under 3 years of age in order to find a tool to monitor BMD in children without the use of expensive imaging techniques. One-way ANOVA and chi-square tests were used to determine the associations of age and serum trace elements with BMD. Multivariable logistic regression analysis was used to test the correlation of five serum trace elements with BMD after adjustments for potential confounding factors in children under 3 years of age. Significant associations between age and four serum trace elements and BMD were found. Compared to the group with the lowest serum levels detected, the adjusted odds ratio (OR) for the incidence of normal bone mineral density in the third magnesium concentration tertile, the third iron concentration tertile, the fifth copper concentration quintile, the third zinc concentration quintile, and the fifth zinc concentration quintile were 1.30 (95% confidence interval (CI) 1.02–1.67), 1.43 (95% CI 1.11–1.84), 1.42 (95% CI 1.04–1.94), 1.46 (95% CI 1.05–2.04), and 1.48 (95% CI 1.06–2.06), respectively. However, there was no significant correlation between serum lead level and BMD in this study. Age and serum magnesium, iron, copper, and zinc levels are positively associated with BMD in children under 3 years old.

## Introduction

Children are at the most rapid stage of growth and development, and healthy bone physiology can help fight bone disease and reduce the risk of fractures in later adulthood. Bone health is closely related to normal bone mass, which increases quickly. The available tools to evaluate bone mass in children are limited, however bone density is an important indicator of bone mass. Bone mineral density (BMD) is defined as the ratio of the amount of mineral to bone volume (g/cm^3^) and more than 85% of peak bone mineral density is accumulated at the age of 18, especially during adolescence^1,2^. Osteoporosis results from decreased BMD and affects more than 200 million people worldwide. Monitoring BMD during the rapid growth and development of early childhood is important to optimise bone healthing later life.

BMD of children is related to many factors, including genetic factors, intrauterine environmental factors^3^, postpartum nutritional factors, and behavioural factors^4^. Trace elements are one of important nutrients in the postpartum nutritional factors. Trace elements serve as coenzymes, parts of hormones and vitamins, and important regulators of signal transduction, gene expression, and blood transportation. They also function as structural components of the skeletal system, teeth, skin, and hair^5^. A consensus has been reached on the importance of calcium in maintaining bone health^6^. However, other trace elements such as magnesium, iron, copper, zinc, etc. are also important for bone metabolism and BMD^7^. Associations between BMD and trace elements in children with obesity, childhood Wilson's disease, childhood burns, and thalassemia have been reported and these studies also proved the intake of trace elements deficiencies can correct BMD^8–11^.

Monitoring the BMD in children is important to evaluate the health and development of bones in children and prevent osteoporosis in adults. To our knowledge, no direct, large sample study on the link between the serum levels of trace elements and BMD in children under 3 years old has been reported. We performed a cross-sectional study to determine the association between BMD and trace elements in children under the age of 3 years in order to prevent possibility of suffering from low bone density when adulthood is reached. The results of this study can be used as a tool to monitor BMD in children without the use of expensive imaging techniques.

## Results

### Statistical description of basic data

The participants were divided into a normal BMD group and a low BMD group. The average age of the low BMD group was 9.02 ± 7.28 months and 61.01% were boys. The mean concentrations of serum magnesium, iron, lead, copper, and zinc in the low BMD group were 1.54 ± 0.20 mmol/L, 8.25 ± 0.83 mmol/L, 31.97 ± 8.00 μg/L, 25.01 ± 5.25 μmol/L, and 81.11 ± 13.29 μg/dL, respectively. Age, gender, body length, weight, body-length-for-age Z Scores, Weight-for-age Z scores, head circumference, serum magnesium, iron, lead, copper, and zinc, and bone mineral density speed of sound (SOS) measurements were all significantly lower in the low BMD group compared to the normal BMD group (Table 1).

**Table 1:** Participant characteristics.

	Normal BMD group	Low BMD group	P
Participants, n (%)	631 (26.16)	1781 (73.84)	–
Age (months)	16.31 ± 9.06	9.02 ± 7.28	< 0.005
Boys (%)	61.01	51.88	< 0.005
Body length (cm)	78.73 ± 9.72	70.21 ± 9.00	< 0.005
Weight (kg)	10.32 ± 2.26	8.68 ± 2.32	< 0.005
Height-for-Age Z scores	0.12 ± 1.06	0.29 ± 1.11	< 0.005
Weight-for-Age Z scores	0.16 ± 0.95	0.51 ± 1.06	< 0.005
Head circumference (cm)	45.74 ± 3.37	43.45 ± 3.08	< 0.005
SOS (m/s)	3297.20 ± 159.07	2997.06 ± 165.66	< 0.005
Mg (mmol/L)	1.57 ± 0.20	1.54 ± 0.20	0.02
Fe (mmol/L)	8.49 ± 0.90	8.25 ± 0.83	< 0.005
Pb (μg/L)	33.88 ± 7.83	31.97 ± 8.00	< 0.005
Cu (μmol/L)	25.61 ± 5.51	25.01 ± 5.25	0.02
Zn (μg/dL)	85.70 ± 12.93	81.11 ± 13.29	< 0.005

Categorical variables are shown as average (percentage). Continuous variables are shown as mean ± standard deviation.

*n* number, *BMD* bone mineral density, *SOS* speed of sound, *Mg* magnesium, *Fe* iron, *Pb* lead, *Cu* copper, *Zn* zinc.

### Association between age and BMD

**Figure 1:**
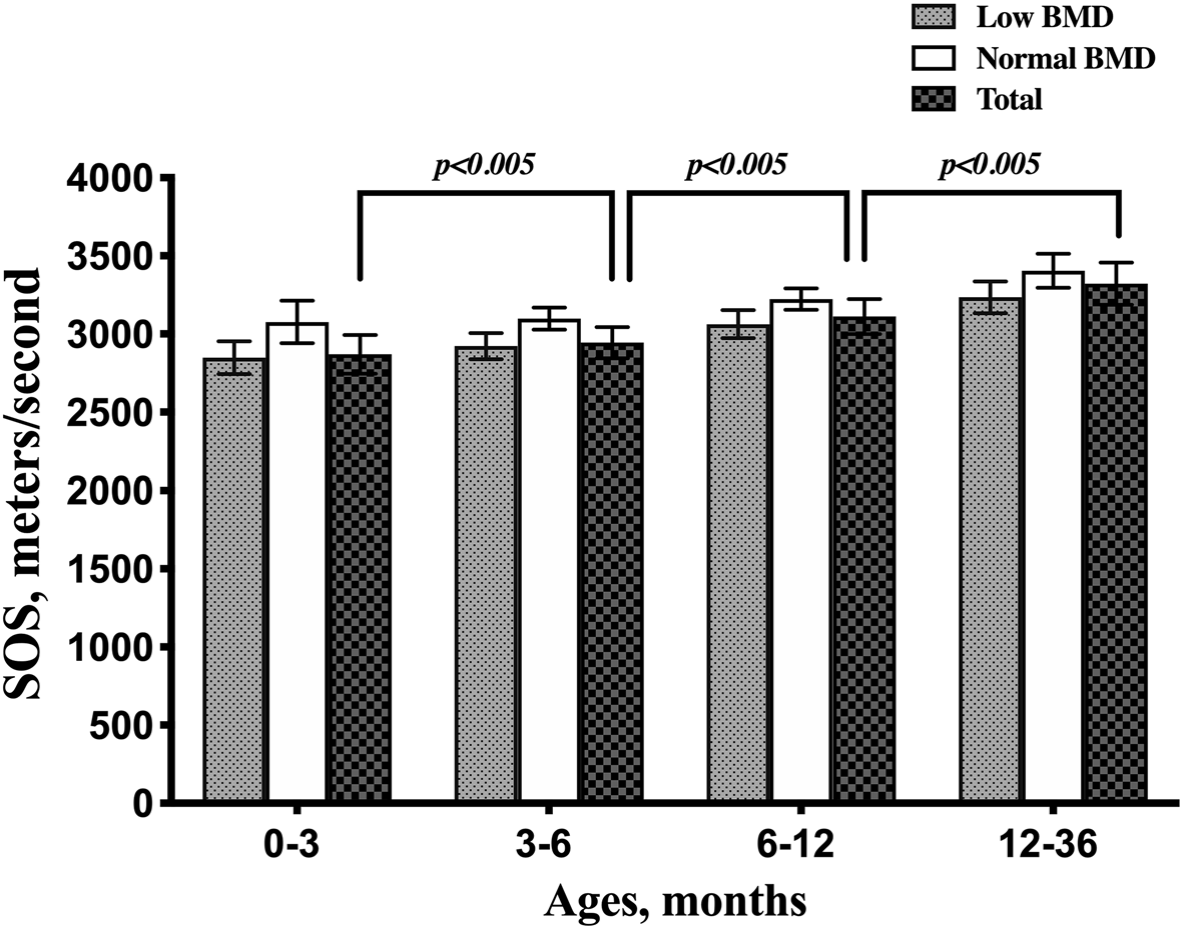
Gradual increase of mean SOS values measured at both normal BMD(white bars) , low BMD (grey bars) and total participants(black bars) with ages progressing. Error bars represent 1 standard deviation from the mean. *BMD, bone mineral density; SOS, speed of sound.*

Participants aged 0–3 months had a mean SOS value of 2867.58 ± 124.48 m/s and 91.73% of these participants had low BMD. Participants aged 3–6 months had a mean SOS value of 2942.64 ± 99.55 m/s and 88.25% of these participants had low BMD. Participants aged 6–12 months had a mean SOS value of 3109.83 ± 111.75 m/s and 70.44% of these participants had low BMD. Participants aged 12–36 months had a mean SOS value of 3319.49 ± 135.19 m/s and 50.00% of these participants had a low BMD. The mean SOS values and incidence of BMD of the four age groups were significantly different (P < 0.005, Table 2). Gradual increase of mean SOS values measured at both normal BMD, low BMD and total participants with ages progressing (Fig. 1).

**Table 2:** Comparison of speed of sound and bone mineral density in different age groups.

	0–3 months	3–6 months	6–12 months	12–36 months	P
Participants (n)	423	749	548	692	
SOS, meters/second	2867.58 ± 124.48	2942.64 ± 99.55	3109.83 ± 111.75	3319.49 ± 135.19	< 0.005
Normal BMD (%)	35 (8.27)	88 (11.75)	162 (29.56)	346 (50.00)	
Low BMD (%)	388 (91.73)	661 (88.25)	386 (70.44)	346 (50.00)	< 0.005

Categorical variables are shown as average (percentage). Continuous variables are shown as mean ± standard deviation.

*n* number, *BMD* bone mineral density, *SOS* speed of sound.

### Association between serum levels of trace elements and BMD

Serum levels of magnesium, iron, zinc, and copper were positively correlated with BMD, as shown in Table 3. The serum level of lead was not found to be associated with the incidence of normal BMD.

**Table 3:** Association between serum levels of trace elements and bone mineral density.

	Median concentration	Participants (n)	Normal BMD (%)	Unadjusted OR (95% CI)	P values	Multivariable-adjusted OR (95% CI)	P
**Mg (mmol/L)**							
T1 ≤ 1.45 (lowest)	1.35	809	23.0	1.00 (reference)	–	1.00 (reference)	–
T2 1.46–1.64	1.55	808	26.6	1.21 (0.97, 1.52)	0.09	1.18 (0.92, 1.52)	0.18
T3 ≥ 1.65 (highest)	1.75	795	28.9	1.36 (1.09, 1.71)	0.01	1.30 (1.02, 1.67)	0.04
P for trend				0.01		0.04	
**Fe (mmol/L)**							
T1 ≤ 7.73 (lowest)	7.43	800	20.3	1.00 (reference)	–	1.00 (reference)	–
T2 7.74–8.59	8.13	809	24.5	1.28 (1.01, 1.62)	0.04	1.04 (0.81, 1.35)	0.75
T3 ≥ 8.60 (highest)	9.22	803	33.7	2.01 (1.60, 2.52)	< 0.005	1.43 (1.11, 1.84)	0.01
P for trend				< 0.005		< 0.005	
**Pb (μg/L)**							
T1 ≤ 30.00 (lowest)	24.60	813	20.9	1.00 (reference)	–	1.00 (reference)	–
T2 30.1–36.6	33.65	796	25.3	1.28 (1.01, 1.61)	0.04	1.15 (0.89, 1.48)	0.29
T3 ≥ 36.70 (highest)	40.00	803	32.4	1.81 (1.45, 2.27)	< 0.005	1.13 (0.88, 1.45)	0.36
P for trend				< 0.005		0.34	
**Cu (μmol/L)**							
T1 ≤ 20.49 (lowest)	18.27	495	23.0	1.00 (reference)	–	1.00 (reference)	–
T2 20.53–23.56	22.19	466	24.7	1.10 (0.81, 1.47)	0.55	1.07 (0.77, 1.48)	0.79
T3 23.61–26.35	25.12	496	25.6	1.15 (0.86, 1.54)	0.35	1.07 (0.78, 1.48)	0.66
T4 26.36–29.75	28.30	472	27.1	1.24 (0.93, 1.67)	0.14	1.11 (0.80, 1.53)	0.53
T5 ≥ 29.76 (highest)	32.20	483	30.4	1.46 (1.01, 1.95)	0.01	1.42 (1.04, 1.94)	0.03
P for trend				0.01		0.03	
**Zn (μg/dL)**							
T1 ≤ 71.06 (lowest)	63.05	481	17.3	1.00 (reference)	–	1.00 (reference)	–
T2 71.08–80.11	75.79	483	22.6	1.40 (1.02, 1.92)	0.04	1.32 (0.94, 1.86)	0.11
T3 80.12–86.67	83.32	482	28.4	1.90 (1.40, 2.59)	< 0.005	1.46 (1.05, 2.04)	0.03
T4 86.71–94.21	90.24	481	26.6	1.74 (1.27, 2.37)	< 0.005	1.28 (0.92, 1.80)	0.15
T5 ≥ 94.25 (highest)	98.28	485	35.9	2.68 (1.99, 3.62)	< 0.005	1.48 (1.06, 2.06)	0.02
P for trend				< 0.005		0.04	

Multivariable model was adjusted for age (continuous data), gender (male, female), body length (continuous data), weight (continuous data) and head circumference (continuous data).

*n* number, *BMD* bone mineral density, *Mg* magnesium, *Fe* iron, *Pb* lead, *Cu* copper, *Zn* zinc, *OR* odds ratio, *CI* confidence interval.

The incidence of normal BMD in the group with the highest serum magnesium concentration was 28.9%, which was significantly higher than the incidence of normal BMD in the group with the lowest serum magnesium concentration (23.0%) (unadjusted odds ratio (OR) 1.36, 95% confidence interval (CI) 1.09–1.71, P = 0.01, P for trend = 0.01; multivariate adjusted OR 1.30, 95% CI 1.02–1.67, P = 0.04; P for trend = 0.04).

The incidence of normal bone mineral density in the group with the highest serum iron concentration was 33.7%, which was significantly higher than the incidence of normal BMD in the group with the lowest iron concentration (20.3%) (unadjusted OR 2.01, 95% CI 1.60–2.52, P < 0.005, P for trend < 0.005; multivariate adjusted OR 1.43, 95% CI 1.11–1.84, P = 0.01; P for trend < 0.005).

The incidence of normal BMD in the group with the highest serum zinc concentration was 35.9%, which was significantly higher than the incidence of normal BMD in the group with the lowest zinc concentration (17.3%) (unadjusted OR 2.68, 95% CI 1.99–3.62, P < 0.005, P for trend < 0.005; multivariate adjusted OR 1.48, 95% CI 1.06–2.06, P = 0.02; P for trend = 0.04). The incidence of normal BMD in the group with the third serum zinc concentration was 28.4%, which was significantly higher than the incidence of normal BMD in the group with the lowest zinc concentration (17.3%) (unadjusted OR 1.90, 95% CI 1.40–2.59, P < 0.005, P for trend < 0.005; multivariate adjusted OR 1.46, 95% CI 1.05–2.04, P = 0.03; P for trend = 0.04).

The incidence of normal bone mineral density in the group with the highest serum copper concentration was 30.4%, which was significantly higher than the incidence of normal BMD in the group with the lowest serum copper concentration (23.0%) (unadjusted OR 1.46, 95% CI 1.01–1.95, P = 0.01, P for trend = 0.01; multivariate adjusted OR 1.42, 95% CI 1.04–1.94, P = 0.03; P for trend = 0.03).

## Discussion

We found that the serum levels of iron, magnesium, zinc, and copper are positively associated with BMD in children under 3 years old.

Bone mineral density is the degree of the compactness of bone, which reflects the mineral content per area, development, and texture of the bone. Regardless of gender, BMD increases during childhood and this increase becomes even greater during adolescence^12^. The BMD of the proximal femur peaks at approximately 20 years of age and the overall BMD peaks 6–10 years later^13^. Approximately 90% of BMD is accumulated before 20 years of age^14^. The BMD in children is used to determine a population’s risk of developing osteoporosis, monitor and prevent fragile fractures, and observe the influential factors of hypogonadism^1,15^. Osteoporosis is associated with many primary and secondary paediatric diseases^16,17^. Studies have shown that low BMD during adolescence can result in permanent low BMD in adulthood^18^. The vital period for the growth and development of children is between zero and 3 years of age. Children grow rapidly and actively metabolize during this period. Studies on the change of BMD and its influential factors can identify favourable and adverse factors, which will help maintain children's bone health, predict the risk of osteoporosis in adults, and reduce the occurrence of bone-related diseases.

Although the aetiology is unclear, many factors may play a role in abnormal bone mineral density, including genetics, sex, age, height and weight, trace elements, and behavioural factors. Our results indicate that low BMD is common in children under 3 years old in this area. The incidence of low BMD was highest in children aged 0–3 months in this study. This result is consistent with the results of Natascia Di lorgi’s review^1^. The growth and development of children is closely related to BMD. Stunting was defined as height-for-age Z scores below − 2.0 and underweight was defined as weight-for-age Z scores below − 2.0^19^. Our results indicate that low BMD in this area has nothing to do with the growth and development situation and nutrition. Low BMD in children under 3 years old may have several explanations. First, children grow quickly, resulting in a very high nutrient demand. Second, the feeding mode of infants may account for low BMD. Studies have shown that children who are exclusively breastfed have a lower rate of bone deposition than those who are fed formula^20^. Third, low BMD may be related to the activity time of infants and children. Younger children have longer sleep time and shorter exercise time than older children. Studies have shown that physical exercise before adolescence has an impact on BMD, especially the BMD of the lower limbs^21^. Lastly, the fact that young children typically spend more time indoors may account for lower BMD. The sun’s ultraviolet rays are closely related to the regulation of calcium and phosphorus and the production of active vitamin D.

Calcium has been proven to be associated with BMD. It plays a vital role in several biological functions, such as muscle contraction, nerve conduction, mitosis, and structural support of the skeleton^22,23^. Serum calcium has been verified to stimulate acute responses in osteoblasts and osteoclasts. Calcium’s role in bone metabolism is well established, therefore, calcium was not included in this study.

Iron is also closely related to bone metabolism. Iron acts as a coenzyme to activate 2-oxoglutarate-dependent dioxygenase, supporting collagen synthesis, and activates 25-hydroxy-cholecalciferol hydroxylase, promoting bone mineralization^24^. In an animal studies, bone volume fraction (bone volume/total volume), trabecular number, and thickness were reduced in iron-deficient rats with lower BMD than control rats^25^. Human studies have suggested similar results. Pre-menopausal women with iron deficiency anaemia have been reported to have higher levels of bone resorption markers and lower levels of bone formation markers^26^. Similarly, children with thalassemia have been reported to have lower lumbar and hip BMD than healthy children^27^. These reported results are consistent with the results of our study. We found that children with a normal BMD have higher serum levels of iron than children with low BMD and that the incidence of normal BMD increases as the serum level of iron increases.

Magnesium is an essential component of apatite crystals and is released following bone resorption. Approximately 60% of the body’s magnesium is stored in the skeleton. Magnesium can be found on cortical bone and contributes to the production of hydroxyapatite^28^. Studies have shown that magnesium deficiency may reduce osteoblastic activity, due to a decrease in the osteoblastic biomarkers alkaline phosphatase and osteocalcin^29,30^. Also, increased osteoclastic functions have been observed in a state of magnesium deficiency^31^. Magnesium deficiency affects calcium homeostasis by affecting parathyroid hormone (PTH) and 1,25(OH)_2_-vitamin D, indirectly affecting BMD^32–35^. Clinical studies have shown that serum levels of magnesium are positively associated with calcaneal bone ultrasound analysis of women and negatively associated with the risk of fracture in both men and women^36^. Magnesium deficiency contributes to osteoporosis and osteoarthritis^37,38^. In this study, we found that participants with normal BMD have higher serum levels of magnesium than participants with low BMD and that the incidence of normal BMD increases as the serum level of magnesium increases.

Zinc is a vital coenzyme that plays a role in DNA, RNA, and protein synthesis. Zinc is involved in the activity of osteoblasts, synthesis of collagen, and inhibition of osteoclastic bone resorption^39^. Animal studies have reported that zinc may help prevent osteoporosis and may play a protective role during osteoporosis therapy^40^. Clinical studies have indicated that zinc supplementation can improve the total bone mass in young patients with thalassemia^41^. Serum zinc levels have been positively associated with osteocalcin in healthy adults^42^. Zinc has been reported to activate RUNX2 via the cAMP-PKA-CREB signalling pathway, which has osteogenic impacts on hBMSCs^43^. Zinc is essential for bone mineral density during childhood, as one study reported decreased somatomedin IGF-1 in zinc-deficient children^44^. We found that children with normal BMD have higher serum levels of zinc than children with low BMD and that the incidence of normal BMD increases as the serum level of zinc increases.

Copper also acts as a coenzyme and plays a role in regulating bone growth and development. Studies have suggested that the activation of lysyl oxidase, a copper-containing oxygenase, induces the formation of collagen and elastin crosslinking^45,46^. Copper deficiency reduces the activity of antioxidant enzymes and superoxide dismutase, increasing free radical production while decreasing the activity of osteoclasts and osteoblasts^47,48^. Copper deficiency rarely occurs in humans. One study reported that adults with severe tooth wear and decreased enamel have decreased copper levels and spinal BMD^49^. Several studies have shown that copper deficiency in children can lead to metabolic bone diseases, including osteoporosis^50^. We found that children with normal BMD have higher serum levels of copper than children with low BMD and that the incidence of normal BMD increases as the serum level of copper increases.

Lead exposure can lead to abnormal bone mineral density^51^. The accumulation of lead in bone causes osteoblastic dysfunction and inhibited matrix production^52^. Studies on animals have shown that lead exposure results in decreased BMD^53^, bone accrual, and bone strength in mice^54^. The cortices in distal tibias and bone mineral density were associated with lead content in women^55^ and a cohort study showed that lead exposure in childhood negatively affects bone mineral density^56^. However, our data show that the serum lead level in participants with a normal BMD was significantly higher than that in participants with a low BMD, and we found no significant difference in the incidence of normal BMD among groups with graded lead levels. This may be due to the fact that lead is absorbed by the blood and stored in mineralized tissues and that bones account for more than 75% of the lead load during childhood^57^. Lead exposure is usually associated with specific occupations or environments.

To our knowledge, this is the first report of the direct association of serum trace elements and BMD in a large sample of participants under 3 years old. The results of this study provide a new perspective to paediatric health care guidance and education. However, this study is not without limitations. SOS *Z*-scores were calculated according to the normative data derived from a sex- and age- matched Asian population, provided by the manufacturer and there is an error between the results and the actual situation of Chinese race. The cross-sectional design excludes causality, thus future prospective studies should be conducted to establish causality between serum trace elements and BMD in children. The relationship between lead and BMD found in this study is partially inconsistent with several existing studies. A more accurate approach to measure serum trace elements may help clarify these inconsistencies. Though we used a multivariate logistic regression model to account for several confounding factors, there may be additional paediatric diseases that were present among our participant population that were not accounted for. More research is required to identify childhood diseases that may affect BMD.

In conclusion, we found that child age and serum levels of magnesium, iron, copper, and zinc are positively associated with BMD in children under 3 years old.

## Methods

### Study population

We analysed data from children who underwent routine health examinations at the Department of Health Examination Centre (Open to the general public), Xiangya Hospital, Central South University in Changsha, Hunan Province, China from October 2016 to April 2017. This research was approved by the ethics committee of Xiangya Hospital and conducted according to the Declaration of Helsinki. Because all participants were under 18 years of age, their parents or legal guardians provided informed consent for the use of this data. The inclusion criteria were as follows: under 36 months of age, underwent quantitative ultrasound to measure BMD, and availability of all basic data (age, gender, body length, weight, and head circumference) and all serum levels of trace elements (magnesium, iron, lead, copper, and zinc). We enrolled 2412 participants.

### Assessment of bone mineral density

The quantitative ultrasound bone mineral density system (Sunlight Company, Israel) was used to measure the BMD speed of sound (SOS) using the standard protocol. Two trained sonographers who were blinded to the clinical symptoms of the participants independently evaluated each participant. Ultrasound doctors are only responsible for the operation of bone mineral density and the interpretation of results, and do not know the grouping principle of this experiment. In addition, the two ultrasound doctors do not know each other's measurement results. All inconsistencies were resolved by discussion, and intra-observer coefficients of variation (CVs) were 0.68% in 142 children and BMD measurements were performed by 2 different investigators resulting in interobserver CVs of 1.54% in 95 children. The SOS values of the middle segment of the tibia were measured in children under 2 years old, and the SOS values of the distal radius were measured in children over 2 years old. The standard deviation (Z values) and percentile (P values) of the measured SOS values were calculated and the values the normative data derived from a sex- and age- matched Asian population, provided by the manufacturer. Normal BMD was defined as Z values > 0 or P values > 50. Low BMD was defined as P values ≤ 50.

### Determination of serum trace elements

Approximately 2 ml of blood was collected from the peripheral vein blood of the ring finger after fasting (Participants who were able to overnight fast fasted for at least 4 h. For participants who needed to breastfeeding day and night or were unable to fast overnight, blood tests were recommended for at least 4 h after the first meal in the morning, After the test, we provided food for the participants.). The blood samples were collected into tubes containing heparin and stood for 30 min before being centrifuged for 10 min at 2000×*g*. The samples were stored at 4 °C until they were analysed. Serum magnesium, iron, lead, copper, and zinc levels were obtained using the chemiluminescence approach by spectrum analyser BH7100 (Bohui-Tech, Beijing, China). All tests were performed in strict accordance with the standard protocol of the instrument and kit instructions, and quality control was performed to ensure the accuracy of the results.

### Statistic analysis

Continuous variables are represented as average ± standard deviation, and categorical variables are represented at mean (%) or n (%). The student’s t test was used to determine the differences in continuous variables, and the chi-square test was used to determine the differences in categorical variables. The participants were classified into four age groups: 0–3 months, 3–6 months, 6–12 months, and 12–36 months. One-way ANOVA and LSD-t tests were used to determine the association between SOS values and age, while the correlation between the incidence of normal BMD and age was evaluated using the chi-square test. Serum magnesium, iron, and lead concentrations were divided into three categories according to tertile distribution, while serum copper and zinc concentrations were divided into five categories according to quintile distribution. The odds ratios (ORs) and 95% confidence intervals (CIs) for the association between each concentration of the trace elements and the incidence of normal BMD were calculated for each tertile or quintile, and the lowest tertile or quintile was used as the reference category for each trace element. Multivariable logistic regression was used to evaluate the association between each trace element and the incidence of normal BMD. The variables included age (continuous data), gender (male, female), body length (continuous data), weight (continuous data), and head circumference (continuous data). Height-for-age Z scores and Weight-for-age Z scores were calculated by using the “WHO Child Growth Standards” analysis programs in Epi Info 2002 Software and other analyses were performed using SPSS v25.0 (IBM Corp. Released 2017. IBM SPSS Statistics for Macintosh, Version 25.0. Armonk, NY: IBM Corp.). A P value < 0.05 was considered statistically significant.
